# Surgical treatment of chronic pulmonary aspergillosis using preventive latissimus dorsi muscle flaps

**DOI:** 10.1186/s13019-015-0354-2

**Published:** 2015-11-05

**Authors:** Yoshinobu Hata, Hajime Otsuka, Takashi Makino, Satoshi Koezuka, Keishi Sugino, Nobuyuki Shiraga, Naobumi Tochigi, Kazutoshi Shibuya, Sakae Homma, Akira Iyoda

**Affiliations:** 1 Division of Chest Surgery, Toho University School of Medicine, Tokyo, Japan; 2 Division of Respiratory Medicine, Toho University School of Medicine, Tokyo, Japan; 3 Department of Radiology, Toho University School of Medicine, Tokyo, Japan; 4 Department of Surgical Pathology, Toho University School of Medicine, Tokyo, Japan

**Keywords:** Pulmonary aspergillosis, Surgical treatment, Hemoptysis, Muscle flap, Empyema, Bronchial artery embolization

## Abstract

**Background:**

Surgery for chronic pulmonary aspergillosis is often technically risky. The choice of immediate thoracoplasty or muscle flap plombage to prevent postoperative space problems remains controversial. This study focused on the use of muscle flaps to prevent postoperative complications.

**Methods:**

During an 8-year period (2004 to 2012), all patients surgically treated for chronic pulmonary aspergillosis were enrolled in this retrospective study. Concomitant intrathoracic transposition of the latissimus dorsi muscle flap has been performed since 2011. The clinical records of these patients were reviewed retrospectively.

**Results:**

From 2004 to 2012, 16 patients were treated for chronic pulmonary aspergillosis. Fifteen patients received lobectomies and one had a partial resection. A preventive latissimus dorsi muscle flap was used in 6 patients (37 %). No postoperative deaths occurred. Prolonged air leaks appeared in 2 patients without muscle flaps, resulting in empyema in both. None of the patients with preventive muscle flaps suffered prolonged air leaks and subsequent empyema. In the outpatient clinic, late onset air leaks developed in 2 patients, one of whom had a lobectomy with muscle flap while the other had a lobectomy without muscle flap. Residual pleural space persisted in these two patients and *Aspergillus* infection later recurred.

**Conclusions:**

Concomitant latissimus dorsi muscle flaps may be effective for the prevention of prolonged air leaks and subsequent empyema. Late onset air leaks are problematic.

## Background

Surgery for chronic pulmonary aspergillosis is often technically hazardous, resulting in a complicated postoperative outcome [1–3]. Chronic pulmonary aspergillosis may occur following a pulmonary insult such as tuberculosis, sarcoidosis, or pneumothorax [1, 2, 4]. The presence of hemoptysis is the most commonly used indicator of the need for surgery due to the risk of massive and fatal blood loss [1, 5]. Up to 30 % of patients with minor hemoptysis may subsequently have life-threatening hemoptysis [1, 2, 6]. Dense adhesion, pleural thickening, and incomplete re-expansion of residual lung parenchyma have resulted in mortality rates of up to 43 % and morbidity rates of up to 60 %, characterized by hemorrhage, residual pleural space, bronchopleural fistula, and empyema [1, 7]. The decline of tuberculosis has led to a dramatic reduction in both mortality and morbidity [1, 8–10], but surgical risk remains an issue in complex cases. Surgical treatment has been helpful not only in reducing symptoms but also in prolonging the survival of patients, including those who are asymptomatic [7, 8, 11]. The overall 10-year survival of surgically treated patients (84.8 %) is significantly better than the survival of medically treated patients (56.7 %), even in patients without symptoms (82.5 % vs 50.5 %) [7]. Our institute recently introduced the use of a primary latissimus dorsi muscle flap to prevent prolonged air leaks that result in postoperative pyothorax. In cases with life-threatening hemoptysis, bronchial artery embolization was performed first as a temporizing measure, and non-emergency surgical treatment was planned. Here we report the results of a retrospective study on the surgical treatment of chronic pulmonary aspergillosis, with specific attention on the use of a preventive latissimus dorsi muscle flap.

## Methods

During an 8-year period (2004 to 2012), all patients surgically treated for chronic pulmonary aspergillosis in our institution were enrolled in this retrospective study. The clinical records of these patients were reviewed retrospectively for clinical presentation, underlying disease, preoperative treatment such as anti-fungal therapy or bronchial artery embolization, indications for surgery, surgical procedures performed, postoperative mortality, complications, and long-term follow-up status. A diagnosis of chronic pulmonary aspergillosis was made on the basis of clinical symptoms, radiologic findings and serological examination, such as serum *Aspergillus* precipitating tests or serum beta-D glucan testing. Patients were classified as having simple or complex aspergilloma on the basis of medical imaging and operative findings: simple aspergilloma was defined as a thin-wall cavitation occurring in an otherwise healthy lung, while complex aspergilloma was found either in a thick-walled cavitation or in the presence of severe underlying parenchymal or pleural sequelae [5, 12]. Aspergillomas and *Aspergillus* organisms were histologically confirmed on all resected specimens.

We selected patients for surgery if they presented with aspergilloma or unstable symptoms after antifungal therapy with micafungin or voriconazole. Since 2008, bronchial artery embolization is performed first for patients with hemoptysis, and elective surgery is planned thereafter. Since 2011, concomitant intrathoracic transposition of the latissimus dorsi muscle flap has been performed, including one case with primary thoracoplasty. The Institutional Review Board of our institution approved this retrospective study of surgical treatment for chronic pulmonary aspergillosis (#25–39).

The Pearson chi-square test or Fisher exact test was used to compare categorical and dichotomous variables. Analysis of variance with life tables and Kaplan-Meier curves were used for the analyses of overall survival. Differences between two groups were analyzed using the log-rank test. Statistical significance was assumed to exist at two-tailed *p* values less than 0.05. All statistical analyses were performed using a statistical software package (JMP, version 11.0; SAS Institute, Cary, NC, USA).

## Results

### Clinical characteristics and underlying disease

From 2004 to 2012, of 62 patients diagnosed with chronic pulmonary aspergillosis, 16 were surgically treated for chronic pulmonary aspergillosis at our institution. The study group consisted of 14 male patients and 2 female patients with a mean age of 65 years (range 50–78). Patients with pulmonary aspergillosis had a variety of underlying lung diseases. Only 3 patients had no known underlying lung disease (Table 1). Tuberculosis was the most frequent underlying disease (56 %), followed by chronic obstructive pulmonary disease (COPD) (38 %) and pneumothorax (19 %). Comorbidities other than lung disease were found in all 16 patients. Diabetes mellitus was the most frequent comorbidity (63 %). Each patient had at least one of the following diseases: tuberculosis, COPD or diabetes mellitus. None of the patients had human immunodeficiency virus infection.

**Table 1:** Patient clinical characteristics

Variable	Total	With flap	Without flap	*p*
Age (years), mean ± SD	65 ± 8	65 ± 7	66 ± 9	0.824
Sex (Male/Female)	14/2	6/0	8/2	0.500
Smoking status (Current/Former/Never)	10/2/4	3/0/3	7/2/1	0.108
Smoking index (pack-year), mean ± SD	31 ± 26	18 ± 20	39 ± 27	0.130
Body mass index (<18.5/≥ 18.5)	6/10	3/3	3/7	0.607
Performance status (0/1/2/3)	13/1/2/0	4/1/1	9/0/1	0.309
Underlying lung disorders (Tbc/others/none)	9/4/3	3/1/2	6/3/1	0.498
Tuberculosis	9	3	6	
Chronic obstructive pulmonary disease	6	1	5	
Pneumothorax	3	1	2	
Idiopathic pulmonary fibrosis	1	0	1	
Lung cancer	1	1	0	
None	3	2	1	
Comorbidity (diabetes mellitus/others)	10/6	4/2	6/4	1.000
Diabetes mellitus	10	4	6	
Chronic renal failure	2	1	1	
Others^a^	5	2	3	
None	0	0	0	
Location; RUL/LUL	10/6	3/3	7/3	0.607
Size of fungus ball (cm), mean ± SD	3.4 ± 1.4	3.9 ± 1.9	3.1 ± 1.1	0.262
Type of aspergilloma (simple/complex)	1/15	6/0	9/1	1.000
Preoperative antifungal therapy	13/3	6/0	7/3	0.250
Preoperative bronchial artery embolization	9/7	4/2	5/5	0.633

*SD* standard deviation; ^a^Others = liver cirrhosis, ulcerative colitis with steroid use, Marfan syndrome, dilated cardiomyopathy, and gastric cancer

**Table 2:** Surgical procedures and postoperative clinical courses

No.	Age	Surgical	Blood Loss	Complication		Hospital
	/sex	Procedure	(mL)	(in hospital)	(after discharge)	stay^a^ (days)
1	60	Lobectomy	381	Respiratory failure (requiring home oxygen therapy at discharge)	Pneumonia	16
	/m	with MF + TP				
2	61	Lobectomy	940	-		20
	/m	with MF				
3	74	Lobectomy	457	-		16
	/m	with MF				
4	69	Lobectomy	2225	-		10
	/m	with MF				
5	55	Lobectomy	1491	-	Late onset air leak	14
	/m	with MF			+ Recurrence	
6	68	Lobectomy	608	-		31
	/m	With MF				
7	56	Lobectomy	1500	-	Late onset air leak	18
	/f				+ Recurrence	
8	58	Lobectomy	651	-		10
	/m					
9	71	Lobectomy	715	-		16
	/m					
10	75	Lobectomy	132	-		8
	/m					
11	61	Lobectomy	240	-		10
	/m					
12	67	Lobectomy	855	Respiratory failure (requiring home oxygen		58
	/f			therapy at discharge)		
13	64	Lobectomy	180	-		7
	/m					
14	75	Lobectomy	1021	Prolonged air leak		72
	/m			Empyema		
15	78	Lobectomy	210	-	Pneumonia	30
	/m					
16	50	Partial resection	trace	Prolonged air leak		31
	/m			Empyema		

*m* male, *f* female, *MF* muscle flap, *TP* thoracoplasty, ^a^Hospitalization stay length following surgery

The mean Body Mass Index (BMI) of the group was 18.9 (range, 12.3 to 25.6). Six patients (38 %) were classified as underweight (BMI < 18.5) and 3 patients (19 %) were categorized as severely thin (BMI < 16.0). The mean Smoking Index was 31 pack-years (range, 10 to 100) among 12 smokers (75 %). The mean size of the fungus ball was measured as 3.4 cm (range, 1.5 to 6.1 cm). One patient (6 %) had simple aspergilloma and 15 patients (94 %) had complex aspergillomas. Lesions were located in the right upper lobe in 10 patients and in the left upper lobe in 6 patients. There were no statistically significant differences in patient characteristics between the group surgically treated with muscle flaps (*n* = 6) and without muscle flaps (*n* = 10, Table 1).

### Symptoms and preoperative treatment

Seven patients (44 %) were free of symptoms, but demonstrated radiological lesions with fungus balls. Surgical treatment was chosen for the remaining 9 patients because of massive hemoptysis in 6 patients (38 %), minor hemoptysis in 2 patients (13 %) and pneumothorax in one patient (6 %). Previous antifungal therapy had been administered to 15 patients (94 %). The median interval from initial presentation to surgery was 8.2 months (range, 0.5 to 127.9 months).

Preoperative embolization was performed for 4 patients with massive hemoptysis, 2 patients with minor hemoptysis and 3 asymptomatic patients. It was successful for 5 of the 6 patients (83 %) with massive or minor hemoptysis. Embolization for the 3 asymptomatic patients was performed to reduce intraoperative blood loss. The median interval from preoperative embolization to surgery was 6.0 days (range, 1 to 21 days). Fifteen surgical procedures (94 %) were planned as elective surgeries; emergency surgery was performed for only one of the patients with pneumothorax.

### Surgical procedures

Lobectomies were performed through posterolateral thoracotomies in 15 patients with complex aspergillomas, and partial resection through axilla thoracotomy in one patient with simple aspergilloma. After 2011, concomitant intrathoracic transposition of latissimus dorsi muscle flap was performed in 6 patients (38 %), including one patient with primary thoracoplasty resulting from a large residual pleural space. The mean operative time was 323 min (range, 93 to 625 min). Operative time was longer for patients with muscle flaps (mean 406 min, range, 315 to 625 min) than for patients without muscle flaps (mean 273 min, range, 93 to 392 min, *p* = 0.023, *t*-test, Table 3). Mean intraoperative blood loss was 725 mL (range, trace to 2225 mL). There was no statistical difference between patients with muscle flaps (mean 1017 mL, range, 381 to 2225 mL) and those without muscle flaps (mean 550 mL, range, trace to 1500 mL, *p* = 0.140, *t*-test, Table 3). There was no statistical difference between patients with preoperative embolization (mean 903 mL, range, 180 to 2225 mL) and those without preoperative embolization (mean 497 mL, range, trace to 1500 mL, *p* = 0.192, *t*-test).

**Table 3:** Surgical procedures and postoperative morbidities

Variable	Total	With flap	Without flap	*p*
Surgical procedure (Lobectomy/Partial resection)	15/1	6/0	9/1	1.000
Operative time (minutes), mean ± SD	323 ± 118	406 ± 114	273 ± 92	0.023
Blood loss (mL), mean ± SD	725 ± 604	1017 ± 718	550 ± 481	0.140
Complication in hospital	4/12	1/5	3/7	1.000
Respiratory failure	2/14	1/5	1/9	1.000
(requiring home oxygen therapy at discharge)				
Prolonged air leak & empyema	2/13	0/6	2/7	0.486
Complication after discharge	3/13	2/4	1/9	0.518
Pneumonia	1/15	1/5	0/10	0.375
Late onset air leak + Recurrence	2/14	1/5	1/9	1.000
Hospital stay^a^ (days), mean ± SD	22 ± 18	17 ± 7	26 ± 22	0.408

^a^Hospitalization stay length after the surgery

**Table 4:** Recent literature concerning surgically treated chronic pulmonary aspergillosis

Author	Published year	n	Mortality	Morbidity	Air leak	Empyema	Recurrence
Babatasi [15]	2000	84	4 %	69 %	37 %	6 %	0 %
Regnard [5]	2000	89	6 %	42 %	10 %	8 %	0 %
Endo [14]	2001	10	0 %	40 %	ND	10 %	10 %
Park [10]	2002	110	1 %	24 %	ND	12 %	0 %
Kim [11]	2005	88	1 %	27 %	13 %	2 %	5 %
Akbari [9]	2005	60	2 %	33 %	7 %	7 %	0 %
Brik [23]	2008	42	2 %	29 %	2 %	7 %	0 %
Lee [7]	2009	135	4 %	30 %	10 %	2 %	4 %
Lejay [8]	2011	33	0 %	12 %	9 %	3 %	ND
Chen [16]	2012	256	1 %	16 %	3 %	1 %	1 %
Farid [17]	2013	30	0 %	ND	23 %	20 %	26 %
Present cases		16	0 %	25 %	19 %	13 %	13 %
(with MF)		(6)	(0 %)	(17 %)	(0 %)	(0 %)	(17 %)

*MF* muscle flap, *ND* not described

Intraoperative spillage occurred in two patients (Cases 4 and 5 in Table 2). Massive irrigation was performed (6 L and 10 L, respectively) and postoperative antifungal therapy was continued. One patient suffered late onset air leak and recurrent aspergillus infection 22 months following surgery. The other remained free from recurrence 25 months after surgery.

### Postoperative mortality and complications

No postoperative deaths occurred within 30 days. While the postoperative courses of 12 patients (75 %) were uneventful, postoperative nonfatal complications occurred in 4 patients (25 %) (Table 2). Prolonged air leaks developed in 2 patients who had either a lobectomy without the muscle flap or underwent partial resection, resulting in empyema in both. Open window thoracostomy was performed on one patient, and thoracoplasty on the other patient. None of the patients with the preventive latissimus dorsi muscle flap suffered prolonged air leaks and subsequent empyema. While two patients required home oxygen therapy at discharge, all patients were discharged to their homes. No occurrences of postoperative excessive bleeding or bronchial fistula were found. Length of stay in the intensive care unit was 2 days for most patients. One patient did not stay in the intensive care unit, and one patient stayed 3 days. The mean length of hospitalization after surgery was 22 days (range: 7 to 72 days). There was no statistically significant difference in postoperative complications between the two patient groups surgically treated with or without a muscle flap (Table 3).

### Long-term outcomes

**Fig. 1:**
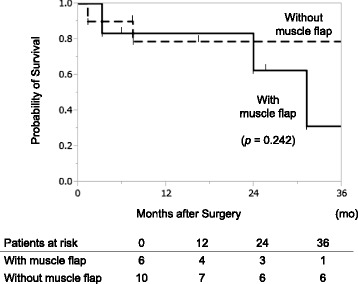
The 3-year overall survival following surgery

In the outpatient clinic, late onset air leaks were found in 2 patients who had received lobectomies, with and without a muscle flap. Two and three months after the surgery, intrathoracic fluid levels decreased with no aspiration pneumonia on chest radiographs. Although no further surgical intervention was required and the patients were clinically stable, residual pleural space persisted and *Aspergillus* infection recurred 8 and 15 months later. One patient declined 24 months after the surgery; the other is alive and well 92 months after the surgery. Two patients suffered pneumonia and were readmitted, and one of them declined 5 weeks after the surgery. The remaining five patients died from malignant disease (*n* = 2), gastric perforation (*n* = 1), and unknown causes (*n* = 2). The 3-year overall survival following surgery was 63 %. There was no significant difference in the 3-year overall survival rates between patients with a muscle flap (31 %) and patients without a muscle flap (79 %, *p* = 0.242, log-rank test, Fig. 1).

## Discussion

We selected patients with chronic aspergillosis for surgery when they presented with aspergilloma or unstable conditions such as hemoptysis after antifungal therapy. For patients with hemoptysis, bronchial artery embolization is performed first and elective surgery is planned thereafter. For patients with complex aspergilloma, who were supposed to have tight adhesions, potential risk for incomplete re-expansions and postoperative persistent air leakage, concomitant intrathoracic transposition of the latissimus dorsi muscle flap was considered. Since full posterolateral thoracotomies were usually performed because of tight adhesion or invasion of a cavitary lesion into the chest wall, the latissimus dorsi was often cut and not available for potential use in a second surgery [3, 11]. While Daly et al. [13] have recommended sparing the latissimus dorsi muscle for potential use, such an approach can hinder the operative field of the first surgery [10]. Harvesting the latissimus dorsi muscle flap at the full posterolateral thoracotomy, instead of cutting or sparing it, and covering the bronchial stump and the cutting surface of the residual lung parenchyma was performed to prevent prolonged air leakage and consequent empyema, although we could not demonstrate the benefit statistically because of the small sample size. Patients with muscle flaps required longer operative time and experienced slightly more blood loss than patients without muscle flaps, but the differences should reflect the difficulty of the surgery. The procedure of harvesting and transposition of the latissimus dorsi muscle flap usually requires approximately 30 min without significant blood loss.

Incomplete reexpansions after lung resections are frequent and responsible for severe complications following surgery for chronic pulmonary aspergillosis [5]. Pleural space problems can manifest as prolonged air leaks, residual pleural pocket, and *Aspergillus* empyema [3]. Before introduction of the latissimus dorsi muscle flap, 2 patients developed prolonged air leaks, which progressed into empyema requiring thoracoplasty in one patient and open window thoracostomy in the other. Prevention of prolonged air leaks is essential for the deterrence of postoperative empyema.

The use of immediate thoracoplasty versus muscle flap plombage to prevent postoperative space problems is still an issue of debate. Massard et al. [3] prefer to use thoracoplasty as a second choice procedure to avoid unnecessary mutilation of some patients. They use the preventive immediate thoracoplasty reported by Personne et al. in up to 25 % of their cases [3]. Akbari et al. [9] and Kim et al. [11] do not employ primary thoracoplasty or latissimus dorsi muscle flaps. Park and Jheon [10], Endo et al. [14, 15] and Babatashi et al. [16] perform concomitant thoracoplasty in some cases to prevent space problems, and the possible development of empyema, if a large volume of dead space is encountered. Chen et al. [17] use additional surgical procedures such as pleural tenting and partial thoracoplasty to avoid prolonged air leaks or residual space, sometimes clamping the phrenic nerve to induce temporary paralysis in patients with good lung function. Farid et al. [18] have reported that persistent space problems might be addressed with a pectoralis flap, modest thoracoplasty, or both, and that the use of a muscle flap reduces the extent of a thoracoplasty, which is helpful for later functioning of the chest.

The late effect of the muscle flap is another concern. Farid et al. [18] explained that initially the muscle flap will often nearly fill the cavity and then atrophy, leaving considerable space. Chest x-rays showed findings of a small finger of muscle, which were believed to be sufficient to keep *Aspergillus* from recolonising the cavity [18]. In our series, although the muscle flap was effective for preventing postoperative persistent air leakage in all cases, one case developed late onset air leakage resulting in persistent space and recurrence of *Aspergillus* infection. Late onset air leakage has also occurred among patients without a preventive muscle flap and resulted in recurrence of *Aspergillus* infection, probably accompanied by pleural aspergillosis. Late onset air leakage and subsequent persistent pleural space would be a possible risk for recurrent *Aspergillus* infections.

While recurrent hemoptysis or *Aspergillus* infection following surgery is reportedly rare [5, 8, 9, 19], Farid et al. [18] found that 8 out of 30 patients (26 %) experienced disease recurrence (Table 4). Although the difference is not obvious, Farid et al. [18] discussed how their postoperative follow-up protocol was formulated to enable early detection of recurrent disease, but concluded that recurrence is problematic. In our institute, 2 of 16 patients (13 %) with late onset persistent air leakage and subsequent pleural space have developed recurrent *Aspergillus* infection. One patient declined 24 months after surgery.

Regarding the extent of resection, anatomical lobectomy might be suitable for inflammatory disease, if the residual lung function permits. Chen et al. [17] preferred complete lobectomy to avoid possible complications and recurrence, with only 2 of 256 cases of postoperative fungal relapse: one patient after a wedge resection by video-assisted thoracic surgery and another after a lobectomy combined with a segmentectomy. Although it is desirable to limit resection as much as possible, to prevent a decrease in lung function [20], a radical resection of affected areas most effectively improves patient outcome [17] and minimizes the risk of postoperative prolonged air leakage. Although division of interlobar fissures using electrocautery has been used to reduce the residual lung volume caused by stapling [10], the potential risk for prolonged air leakage should be considered in cases with incomplete reexpansion of the residual lung.

Preoperative bronchial artery embolization for hemoptysis was successful in 5 out of 6 patients (83 %) in our series, allowing immediate cessation of life threatening hemoptysis. Non-emergency surgical treatment was then planned to proceed within two weeks. This modality has been used as a temporizing measure and not with permanent intent [5, 9, 10, 13, 18]. While the control of bleeding has been achieved in more than 90 % of cases [21], recurrent fatal hemoptysis has been reported in the weeks following embolization [22] or 30-50 % in 3 years [21]. Long delays before surgery are not recommended [5].

Farid et al. [17] reported pre-operative percutaneous endoscopic gastrostomy feeding as another method of preoperative stabilization. Major surgical procedures require sufficient nutritional and performance status [15]. The survival of patients with preoperative weight loss exceeding 10 % of their usual body weight was significantly diminished, with a 5-year actuarial survival rate of only 30 % [5].

This study had several limitations. First, it was a retrospective analysis covering an 8-year period. Second, we could not confirm any statistically significant differences because of the small number of patients. However, we believe this study may provide some useful information for the surgical management of patients with chronic pulmonary aspergillosis.

## Conclusions

In conclusion, concomitant latissimus dorsi muscle flaps may be effective for prevention of prolonged air leaks and subsequent empyema, while late onset air leakage resulting in residual pleural space and recurrent *Aspergillus* infection were problematic. Surgical management remains the mainstay of treatment for aspergilloma. With preoperative stabilization using bronchial arterial embolization, early surgical intervention on a non-emergency basis is recommended.

## Abbreviations

BMI: Body Mass Index
COPD: Chronic obstructive pulmonary disease
